# Bosentan regulates the expression of adhesion molecules on circulating T cells and serum soluble adhesion molecules in systemic sclerosis-associated pulmonary arterial hypertension

**DOI:** 10.1136/ard.2007.080424

**Published:** 2007-11-20

**Authors:** F Iannone, M T Riccardi, S Guiducci, R Bizzoca, M Cinelli, M Matucci-Cerinic, G Lapadula

**Affiliations:** 1 Rheumatology Unit – DIMIMP, University of Bari, Bari, Italy; 2 SOD Internal Medicine 1 and Rheumatology, AOUC Careggi, University of Florence, Florence, Italy

## Abstract

**Objectives:**

To study the expression of adhesion molecules in patients with systemic sclerosis (SSc) with and without pulmonary arterial hypertension (PAH) and the effects of therapy with the endothelin-1 (ET-1) receptor antagonist, bosentan.

**Methods:**

In all, 35 patients with SSc and 25 healthy donors (HD) were selected for this study. Of 35 patients, 10 had isolated PAH assessed by Doppler echocardiography and treated with bosentan. Peripheral blood (PB) lymphocytes were isolated by density gradient centrifugation, and the expression of lymphocyte function-associated antigen-1 (LFA-1), very late antigen-4 (VLA-4) and L-selectin on CD3 T cells was assessed by double immunofluorescence and flow-cytometry. As endothelial activation markers, serum soluble P-selectin, platelet/endothelial cell adhesion molecule (PECAM)-1, vascular cell adhesion molecule (VCAM)-1, intercellular adhesion molecule (ICAM)-1 and von Willebrand factor (vWF) antigen were assessed by ELISA. In patients with SSc-PAH, T cell subsets and soluble endothelial markers were assessed at baseline and after 6 and 12 months of bosentan therapy.

**Results:**

In patients with SSc-PAH, serum soluble ICAM-1, VCAM-1, P-selectin and PECAM-1 levels were higher than in HD at baseline and fell to normal values after 12 months of bosentan therapy. CD3–LFA1 T cells were significantly higher in PAH-SSc at baseline than in HD or SSc and significantly decreased after therapy. CD3–L-selectin T cells were significantly lower in SSc-PAH at baseline than in HD or SSc and rose to normal levels after bosentan therapy.

**Conclusions:**

This study confirms that endothelial activation occurs in SSc, and suggests that changes in the T cell/endothelium interplay take place in SSc-associated PAH. Bosentan seems to be able to hamper these changes and restore T cell functions in these patients.

A crucial role in the pathogenesis of systemic sclerosis (SSc) seems to be played by the interactions occurring between endothelial cells and T cells. Endothelial cells, after metabolic activation, produce an array of cytokines and growth factors and promote immune cells to adhere and migrate through the vessel wall into the extra-vascular space. This leads to fibroblasts and myoblasts activation and changes in extracellular matrix (ECM) remodelling, resulting in an excessive accumulation of ECM components that causes fibrosis of the skin and internal organs and hypertrophy/hyperplasia of the arterial wall of pulmonary and renal vessels.^1^ Circulating peripheral blood T cells adhere to endothelial cells by means of an intricate system of adhesion molecules whose expression increases on the cell surface following activation. Lymphocyte function-associated antigen-1 (LFA-1), very late antigen-4 (VLA-4) and L-selectin are expressed on lymphocytes, while their counter-receptors intercellular adhesion molecule-1 (ICAM-1), vascular cell adhesion molecule-1 (VCAM-1) and CD34/endoglycan are expressed on endothelial cells.^2^ When overexpressed on the activated endothelial layer, VCAM-1, ICAM-1 as well as P-selectin and platelet endothelial cell adhesion molecule-1 (PECAM-1) undergo shedding and their soluble forms, sVCAM-1, sICAM-1, sP-selectin and sPECAM-1, respectively, are detectable in serum and considered to be markers of endothelial cell activity or injury. There is a growing body of evidence that these endothelial activation markers are raised in patients with SSc and that their levels correlate with disease activity and can be regulated by therapy.^3^

Endothelin-1 (ET-1) is a potent mitogenic factor mainly produced by endothelial cells, and exerts its biological activity by interacting with two cell membrane-bound receptors named ET_A_ and ET_B_, expressed on different cells, such as endothelial cells, smooth muscle cells and fibroblasts.^4^ ET-1 has pleiotropic functions, being a potent vasoconstrictor, and stimulates synthesis and accumulation of ECM proteins by fibroblasts and smooth muscle cells. Since its levels have been found to be increased in SSc, it is believed to have a key role in the pathogenesis of this disease.^5^ In particular, ET-1 levels have been detected in lung plexiform lesions in patients with pulmonary arterial hypertension (PAH) and there is evidence from experimental models and clinical studies that ET-1 is directly involved in promoting the remodelling of vessel walls leading to an increase of peripheral vascular resistance and hence a rise in pulmonary arterial pressure.^6^ Recently, a dual endothelin receptor antagonist named bosentan has been shown to be effective and safe for the treatment of patients with SSc with PAH.^7^ In this study, the biological outcome of bosentan on endothelial activity and T cell activation has been evaluated in patients with SSc-associated PAH. Expression of LFA-1, VLA-4 and L-selectin has been investigated on peripheral blood T cells in patients with SSc-PAH at baseline and after bosentan treatment. Serum levels of soluble VCAM-1, ICAM-1, P-selectin, PECAM-1 and von Willebrand factor (vWF) were also assessed.

## METHODS

### Patients

A total of 35 patients with SSc were consecutively recruited at the Rheumatology Unit of the University of Bari, Bari, Italy. Mean age was 51.4 years (range 27–75) and mean disease duration was 10.4 years (range 1–28). All the patients were assigned to the limited cutaneous subset, according to the Leroy subset classification.^8^ A total of 25 healthy donors (HD), coming from the same geographic area and attending the Rheumatology Unit as students, teachers and employers, were enrolled on the basis of their age (mean age 46.7 years, range 25–69). In the patients with SSc, high-resolution chest tomography was carried out to assess interstitial lung fibrosis, as well as respiratory function tests to evaluate pulmonary forced vital capacity (FVC) and alveolar CO diffusion (DLCO), along with full blood count, renal function tests, serum complement levels, liver enzymes, erythrocyte sedimentation rate (ESR). Of 35 patients, 10 had isolated PAH assessed by Doppler echocardiography (tricuspid gradient at rest >35 mmHg), and subsequently confirmed by right heart catheterisation; mean pulmonary artery pressure (mPAP) was 38.6 (9) mmHg, pulmonary vascular resistance was 758 (254) dyne/s/cm, pulmonary capillary wedge pressure was 13 (1.7) mmHg. Patients with SSc-PAH were in New York Heart Association (NYHA) functional classes II–III according to the World Health Organization (WHO) classification, with 6-min walking distance (6-MWD) ranging between 60 and 450 m.^7^ No patient had left ventricular disease at Doppler echocardiography. Further characteristics of the patients with SSc with PAH are shown in detail in table 1. Patients with SSc-PAH were treated with bosentan at the standard dosage of 62.5 mg twice daily for 4 weeks, followed by 125 mg twice daily for 50 weeks. Additional permitted drugs were intestinal prokinetics and H-2 blockers. No patients were on steroids and/or immunosuppressive agents. Clinical assessment, including 6-MWD and Doppler echocardiography, and collection of serum and heparin blood samples were performed at baseline and after 6 and 12 months of bosentan therapy. Written informed consent was obtained from all patients and healthy donors according to the declaration of Helsinki and the study was approved by the Ethics Committee of the Policlinico di Bari.

**Table 1:** Patient characteristics

Parameter	Value
Patients with PAH	10
Age, years	56.4 (31–79)
Disease duration, years	10.2 (2–24)
FVC/DLCO ratio	1.6 (1.37–2.13)
ANA (n)	10
SCL70 (n)	2
ACA (n)	2

Values are mean (range).

ACA, anticentromere antibodies; ANA, antinuclear antibodies; DLCO, CO diffusion; FVC, forced vital capacity; PAH, pulmonary arterial hypertension.

### T lymphocyte phenotype

Peripheral blood (PB) lymphocytes were isolated by gradient density separation on lymphoprep and cell surface antigen expression on T cells was assessed by double immunoflorescence. Briefly, 5×10^5^ cells were incubated with 5 μl of mAb (CD3–PE, CD11a/LFA1-FITC, CD49d/VLA4-FITC, CD62L/L-selectin-FITC, Immunotech, Marseille, France) for 20′ on ice, washed twice and T cell subsets were evaluated by flow cytometry (FACScan, Becton Dickinson, Franklin Lakes, New Jersey, USA). T cell phenotype was evaluated at each timepoint on freshly isolated lymphocytes against a control sample, incubated with rat IgG_1_-FITC/IgG2-PE (DAKO, Glostrup, Denmark).

### Soluble endothelial activation markers

Serum levels of endothelial markers were assessed by commercial ELISA: sICAM-1 ELISA kit (Cellular Communication Investigation, Immunotech, Marseille, France), sVCAM-1 ELISA kit (Immuno Biological Laboratories, Hamburg, Germany), vWF ELISA kit (Gentaur, Brussels, Belgium), sPECAM-1 ELISA kit (Bender MedSystems, Wien, Austria) and sP-selectin ELISA kit (Bender MedSystems), following the manufacturers’ instructions. ELISA experiments were run simultaneously on all samples previously frozen at −30°C.

### Statistical analysis

Values are expressed as mean 1 SD) unless otherwise indicated. Analysis of variance (ANOVA) with the post hoc least square differences (LSD) range test was used to compare the different groups at baseline, whereas a paired Student t test was applied to compare patients with SSc-PAH at the different timepoints. The Mann–Whitney U test was used to compare NYHA functional classes over time. Correlations of clinical data with endothelial activation markers and T cell phenotype were carried out using Pearson or Spearman analysis. The significance level was set at p<0.05.

## RESULTS

### Clinical outcomes

Bosentan treatment significantly improved exercise ability in patients with SSc-PAH with 6-MWD, which rose from 267 (33) m at baseline to 359 (27) m at 6 months and to 376 (40) m at 12 months (p<0.05 vs baseline). Bosentan also induced an improvement of dyspnoea, measured by NYHA, with a significant reduction of the NYHA functional class, observed after 12 months of therapy (median baseline 2.0 vs median 12 months 1.5, p<0.05). PAP values reduced during bosentan treatment, without reaching statistical significance (data not shown). Digital ulcers were present in 8 out of 10 patients and 6 patients did not experience new digital ulcers during bosentan therapy.

### Adhesion molecule expression on T cells

**Figure 1:**
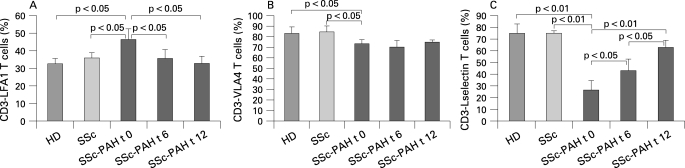
Percentage of peripheral blood T cells expressing lymphocyte function antigen-1 (LFA-1) (A), very late antigen-4 (VLA-4) (B) and L-selectin (C) in healthy donors (HD), patients with systemic sclerosis (SSc) without pulmonary arterial hypertension and patients with SSc with pulmonary arterial hypertension (SSc-PAH) at baseline (t 0), after 6 months (t 6) and 12 months (t 12) of bosentan therapy. Values are mean (SD).

**Figure 2:**
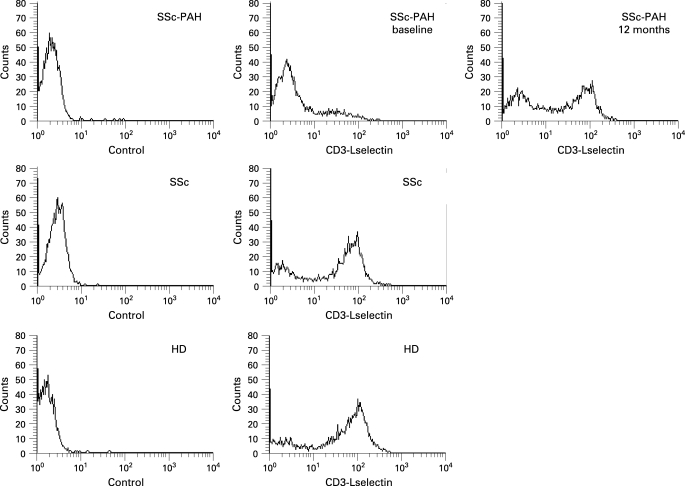
Fluorescence intensity for CD3–L-selectin cell subset from a patient with SSc-PAH at baseline and after 12 months of bosentan therapy (upper panels), from a patient with SSc without PAH (middle panels) and from a healthy donor (lower panels). Negative control histograms (a fluorescent non-binding mAb) are shown on the left. The longitudinal axis shows the percentage of lymphocytes positive for CD3 and L-selectin and the horizontal axis the mean channel fluorescence.

Absolute number of lymphocytes did not change upon bosentan therapy. As shown in fig 1A, PB T cells expressing LFA-1 were significantly higher in patients with SSc-PAH at baseline (mean (SD) 46.3 (6)%) than in HD (32.6 (3)%, p<0.05) or in patients with SSc without PAH (35.8 (5)%, p<0.05). They decreased after 6 months of bosentan therapy (35.5 (5)%, p<0.05 vs baseline) and fell to normal values after 12 months (32.7 (4)%, p<0.05 vs baseline). The proportion of T cells bearing VLA-4 antigen (fig 1B) was significantly reduced in the SSc-PAH group (73.4 (4)%) in comparison with HD (83.3 (6)%, p<0.05) and patients with SSc without PAH (84.8 (5)%, p<0.05), but was not regulated by bosentan treatment after 6 months (70.5 (6)%, not significant vs baseline) nor after 12 months (75.1 (2)%, not significant vs baseline). Expression of L-selectin on T cells (fig 1C) was significantly lower in patients with SSc-PAH (26.4 (8)%) than in the HD group (75.5 (8)%, p<0.01) or in patients with SSc without PAH (75.5 (2)%, p<0.01), and gradually rose following bosentan treatment at the 6 months (43.1 (10)%, p<0.05 vs baseline) and 12 months controls (63.2 (6)%, p<0.01 vs baseline). In fig 2, the representative histograms of the fluorescence intensity for CD3–L-selectin cell subset from a patient with SSc-PAH before and after bosentan therapy, a patient with SSc without PAH and a healthy donor are shown.

### Serum endothelial markers

**Figure 3:**
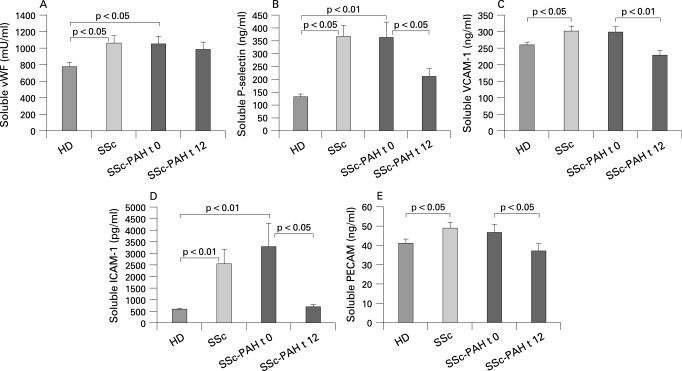
Serum levels of soluble von Willebrand Factor (vWF) (A), P-selectin (B), vascular cell adhesion molecule 1 (VCAM-1) (C), intercellular adhesion molecule-1 (ICAM-1) (D) and platelet endothelial cell adhesion molecule-1 (PECAM-1) (E) in healthy donors (HD), patients with systemic sclerosis (SSc) without pulmonary arterial hypertension and patients with SSc with pulmonary arterial hypertension (SSc-PAH) at baseline (t 0) and after 12 months (t 12) of bosentan therapy. Values are mean SD).

Serum levels of soluble vWF (fig 3A) were increased in patients with SSc-PAH (1043 (97) mU/ml) and patients with SSc without PAH (1061 (86) mU/ml) in comparison with the HD group (770 (54) mU/ml, p<0.05) and did not change after 12 months of bosentan therapy (980 (89) mU/ml, not significant vs baseline). Serum levels of sP-selectin (fig 3B) were significantly higher in the SSc-PAH group (367 (42) ng/ml) and in patients with SSc without PAH (362 (61) ng/ml) than in HD (132 (12) ng/ml, p<0.01), and significantly decreased after 12 months of bosentan therapy (211 (31) ng/ml, p<0.05, vs baseline). Soluble PECAM serum levels were similar in patients with SSc-PAH (48.7 (3) ng/ml), in patients with SSc without PAH (46.7 (4) ng/ml) and in HD (41.1 (2) ng/ml) and were not modulated by bosentan treatment (37.7 (4) ng/ml). Serum levels of sVCAM-1 (fig 3C) were significantly higher in patients with SSc without PAH (300 (16) ng/ml) than in the HD group (261 (9) ng/ml, p<0.05), and were also increased in patients with SSc-PAH (303 (3) ng/ml) but without reaching statistical significance. However, sVCAM-1 levels significantly dropped after 12 weeks of bosentan therapy (229 (15) ng/ml, p<0.01 vs baseline). Furthermore, sICAM-1 serum levels (fig 3D) were significantly higher in patients with SSc-PAH (2536 (647) pg/ml) and patients with SSc without PAH (3293 (1006) pg/ml) than in HD (588 (48) pg/ml, p<0.01), and declined to normal levels following bosentan treatment (696 (98) pg/ml, p<0.05 vs baseline). Finally, soluble PECAM levels (fig 3E) were increased in patients with SSc-PAH (46.7 (4) ng/ml) in comparison with HD (41 (2) ng/ml), but the difference was not statistically significant and significantly reduced after bosentan treatment (37 (3) ng/ml; p<0.05).

No significant correlations were found between adhesion molecules, either soluble or expressed on T cells, and clinical parameters such as modified Rodnan skin score, 6-MWD, NYHA classes, new digital ulcers, FVC and DLCO. Furthermore, no correlations between adhesion molecule changes over time and treatment response were detected.

## DISCUSSION

The pathophysiology of SSc is multifaceted and different types of cells, such as fibroblasts, immune cells, inflammatory cells, endothelial cells, smooth muscle cells, pericytes, myointimal cells, are involved. Each disease picture is unique because the pathological process may lead to different tissue damage depending on the organ involved.^1^ In recent years, the study of pulmonary vasculature involvement has posed a strong challenge since PAH dramatically affects the clinical outcome and survival of patients with SSc, especially those classified within the limited cutaneous subset.^9^

A widely accepted hypothesis is that, maybe on a genetic basis and following unknown external stimuli, endothelial cells and lymphocytes become activated and stimulate fibroblasts and smooth muscle cells to proliferate and produce collagen and other ECM components.^1^ ^10^ Endothelial damage would let leucocytes migrate through the vessels into the extra vascular tissue and promote the activation of the innate immunity. This local inflammatory response would be self limiting without the transition to the adoptive immunity with activation of T cells that sustain a chronic inflammatory state and stimulate fibrosis by directly interacting with fibroblasts and producing fibrogenic mediators, such as interleukin (IL)4, IL13 and transforming growth factor (TGF)-β1.^1^ Focusing on the interplays between endothelial cells and lymphocytes may be crucial to unravel the early events occurring in SSc pathogenesis. Circulating T cells interact with endothelial layer through an intricate system of receptors and their ligands, generally known as “adhesion molecules”.^2^ L-selectin and P-selectin, oligosaccharide proteins belonging to the family of selectins, are expressed on T lymphocytes and endothelial cells, respectively, and reciprocally interact to allow loose adhesion of circulating lymphocytes. If a firm adhesion, which preludes migration through the vessels into the extra-vascular space, occurs, endothelial cells and T cells undergo activation and modulate the expression of their surface receptors.^11^ ICAM-1 and VCAM-1 are upregulated on activated endothelial cells and interact with LFA-1 and VLA-4, respectively, expressed on activated T cells. When overexpressed on the cell surface, some of these proteins are shed in biological fluids as a regulatory mechanism, and the soluble forms, sVCAM-1, sICAM-1 and sP-selectin, can be dosed and used as markers of endothelial cell activation. Many authors have shown that serum activation markers are increased in patients with SSc, and have attempted to correlate them to disease activity.^3^ ^12^^–^19

Among the array of cytokines involved, ET-1, mainly released by endothelial cells, has been shown to play a physiological role in controlling vascular tone by regulating vasodilatation and vasoconstriction. In SSc, changes of crosstalk between ET-1 and its receptors due to a different distribution of ET_A_ and ET_B_ receptors break down this balance, causing vasoconstriction to predominate. Besides vascular tone alterations, ET-1 stimulates smooth muscle cells and myofibroblasts and promote vasoproliferative changes leading to increased pulmonary vasculature resistances and subsequent PAH.^5^ The importance of ET-1 in the pathogenesis of PAH is further corroborated by the beneficial effects of the dual endothelin receptors inhibitor, bosentan, in the treatment of PAH.^7^ ^20^

In this study we evaluated the expression of LFA-1, VLA-4 and L-selectin antigens on circulating T cells and serum levels of soluble vWF, ICAM-1, VCAM-1 and P-selectin in patients with SSc with associated PAH, at baseline and after 12 months of bosentan therapy, as compared to patients with SSc without PAH and healthy subjects. Our results confirm that bosentan treatment improves exercise ability and dyspnoea in patients with SSc-PAH,^7^ and provide further evidence that endothelial activation occurs in SSc, since soluble endothelial activation markers were significantly higher in the whole SSc group than in controls. Interestingly, whereas vWF antigen levels did not change after bosentan therapy, serum levels of sICAM-1, sVCAM-1, sPECAM and sP-selectin fell to normal values following blocking of the ET_A_ and ET_B_ receptors. These findings are apparently in contrast with those recently reported by Sfikakis *et al* who did not detect changes in sICAM-1 levels in patients with SSc following bosentan treatment.^21^ However, the latter was a short-term study in which sICAM-1 was assessed in 10 patients with SSc treated with bosentan at lower dosage for 4 months.

The behaviour of T cell subsets bearing adhesion molecules was noteworthy. T cells expressing LFA-1, the counter-receptor of ICAM-1, were significantly and selectively enhanced in the SSc-PAH group at baseline, and LFA-1 expression was downregulated by bosentan therapy. The reduction of VLA-4 on T cells from patients with SSc-PAH was unforeseen as its expression is expected to increase on activated T cells and the meaning of these findings is unknown. VLA-4 has pleiotropic functions and it is involved in T cells/endothelial cells interactions as well as in T cells/stromal cells interplay. The finding that VLA-4 was selectively reduced on peripheral T cells from patients with SSc-PAH but not in patients with SSc without PAH and HD, suggests that this molecule may be directly involved in SSc-PAH pathogenesis. However, since bosentan did not modify VLA-4 expression on T cells, it is conceivable that ET-1 does not regulate VLA-4. The pattern of L-selectin was remarkable in that its expression on T cells was lower in patients with SSc-PAH than in healthy donors or patients with SSc without PAH, and strikingly increased to normal values after bosentan treatment. These data suggest that bosentan, a dual endothelin receptor antagonist approved for the therapy of PAH, is able to restore T cell functions in patients with SSc with PAH in vivo. We can speculate that blocking both ET-1 receptors can antagonise the detrimental effects of the imbalance of the ET-1 system in SSc. How inhibition of ET-1 receptors may regulate LFA-1 and L-selectin expression on circulating T cells can only be a matter of speculation at the moment. This may be indirectly mediated by the microvasculature, which expresses ET_A_ and ET_B_ receptors, and changes of adhesion molecules on endothelial cells might modify the expression of their respective ligands on circulating T cells. Additionally, it may be assumed that T cells may bear ET-1 receptors on their membrane and that the changes of LFA-1 and L-selectin expression we detected in our patients with SSc-PAH are due to downstream signals following direct stimulation by ET-1, subsequently inhibited by bosentan. This hypothesis needs to be verified by further studies in vitro addressed to show the expression of ET-1 receptors by T cells and the role of ET-1 in modulating T cell phenotype. However, an analogous mechanism has already been shown on human neutrophils. ET-1 downregulated the expression of L-selectin and upregulated the expression of LFA-2 on neutrophils and these effects were selectively prevented by a specific ET_A_ receptor antagonist.^22^ These data seem to be consistent with our results and coherent with the sequential two-step action of these molecules. Resting leucocytes highly express L-selectin and loosely adhere to endothelial cells, the so-called “tethering”, and roll on the endothelial layer. After activation, endothelial cells upregulate ICAM-1 and leucocytes downregulate L-selectin and simultaneously increase LFA-1 expression. LFA-1/ICAM-1 interactions would lead leucocytes to firmly adhere and migrate through the vessels. Furthermore, LFA-1/ICAM-1 ligation may be involved in T cells/fibroblasts interactions. In SSc-PAH, ET-1 could stimulate these events on T cells, which become activated, with high LFA-1 and low L-selectin expression, and migrate in the extra-vascular space, while bosentan down-modulates these processes and prevents further priming of circulating naïve T cells. However, it should be taken into account that dermal fibroblasts may be a further source of increased serum levels of adhesion molecules. ICAM-1 expression on surface fibroblasts is modulated by ET-1 and inflammatory cytokines, is involved in cell–cell and cell–matrix interactions and plays a key role in regulating inflammatory cells binding to dermal tissue.^23^ ^24^

In conclusion, this study provides evidence that ET-1 can induce changes in the T cell/endothelium interplay in SSc-associated PHA and that blocking ET-1 by the administration of bosentan can restore these interactions.

## References

[1] Abraham DJ Varga J Scleroderma: from cell and molecular mechanisms to disease models. Trends Immunol 2005;26:587–95 16168711 10.1016/j.it.2005.09.004

[2] Springer TA Adhesion receptors of the immune system. Nature 1990;346:425–34 1974032 10.1038/346425a0

[3] Kuryliszyn-Moskal A Klimiuk PA Sierakowski S Soluble adhesion molecules (sVCAM-1, sE-selectin), vascular endothelial growth factor (VEGF) and endothelin-1 in patients with systemic sclerosis: relationship to organ systemic involvement. Clin Rheumatol 2005;24:111–6 15349798 10.1007/s10067-004-0987-3

[4] Galie N Manes A Branzi A The endothelin system in pulmonary arterial hypertension. Cardiovasc Res 2004;61:227–37 14736539 10.1016/j.cardiores.2003.11.026

[5] Davenport AP Maguire JJ Endothelin. Handb Exp Pharmacol 2006;295–329. 10.1007/3-540-32967-6_9 16999223

[6] Runo JR Loyd JE Primary pulmonary hypertension. Lancet 2003;361:1533–44 12737878 10.1016/S0140-6736(03)13167-4

[7] Rubin LJ Badesch DB Barst RJ Galie N Black CM Keogh A Bosentan therapy for pulmonary arterial hypertension. N Engl J Med 2002;346:896–903 11907289 10.1056/NEJMoa012212

[8] LeRoy EC Black C Fleischmajer R Jablonska S Krieg T Medsger TA Jr Scleroderma (systemic sclerosis): classification, subsets and pathogenesis. J Rheumatol 1988;15:202–5 3361530

[9] Williams MH Das C Handler CE Akram MR Davar J Denton CP Systemic sclerosis associated pulmonary hypertension: improved survival in the current era. Heart 2006;92:926–32 16339813 10.1136/hrt.2005.069484 PMC1860719

[10] Jimenez SA Derk CT Following the molecular pathways toward an understanding of the pathogenesis of systemic sclerosis. Ann Intern Med 2004;140:37–50 14706971

[11] Sackstein R The lymphocyte homing receptors: gatekeepers of the multistep paradigm. Curr Opin Hematol 2005;12:444–50 16217160 10.1097/01.moh.0000177827.78280.79

[12] Shahin AA Anwar S Elawar AH Sharaf AE Hamid MA Eleinin AA Circulating soluble adhesion molecules in patients with systemic sclerosis: correlation between circulating soluble vascular cell adhesion molecule-1 (sVCAM-1) and impaired left ventricular diastolic function. Rheumatol Int 2000;20:21–4 11149656 10.1007/s002960000072

[13] Ihn H Sato S Fujimoto M Takehara K Tamaki K Increased serum levels of soluble vascular cell adhesion molecule-1 and E-selectin in patients with systemic sclerosis. Br J Rheumatol 1998;37:1188–92 9851267 10.1093/rheumatology/37.11.1188

[14] Denton CP Bickerstaff MC Shiwen X Carulli MT Haskard DO Dubois RM Serial circulating adhesion molecule levels reflect disease severity in systemic sclerosis. Br J Rheumatol 1995;34:1048–54 8542206 10.1093/rheumatology/34.11.1048

[15] Kiener H Graninger W Machold K Aringer M Graninger WB Increased levels of circulating intercellular adhesion molecule-1 in patients with systemic sclerosis. Clin Exp Rheumatol 1994;12:483–7 7842528

[16] Carson CW Beall LD Hunder GG Johnson CM Newman W Serum ELAM-1 is increased in vasculitis, scleroderma, and systemic lupus erythematosus. J Rheumatol 1993;20:809–14 7687701

[17] Blann AD Constans J Carpentier P Renard M Satger B Guerin V Soluble P selectin in systemic sclerosis: relationship with von Willebrand factor, autoantibodies and diffuse or localised/limited disease. Thromb Res 2003;109:203–6 12757775 10.1016/s0049-3848(03)00209-3

[18] Scheja A Akesson A Geborek P Wildt M Wollheim CB Wollheim FA Von Willebrand factor propeptide as a marker of disease activity in systemic sclerosis (scleroderma). Arthritis Res 2001;3:178–82 11299058 10.1186/ar295 PMC30710

[19] Blann AD Illingworth KJ Jayson MI Raised concentrations of von Willebrand factor antigen in systemic sclerosis. Ann Rheum Dis 1991;50:337–8 2042993 10.1136/ard.50.5.337-b PMC1004425

[20] Channick RN Simonneau G Sitbon O Robbins IM Frost A Tapson VF Effects of the dual endothelin-receptor antagonist bosentan in patients with pulmonary hypertension: a randomised placebo-controlled study. Lancet 2001;358:1119–23 11597664 10.1016/S0140-6736(01)06250-X

[21] Sfikakis PP Papamichael C Stamatelopoulos KS Tousoulis D Fragiadaki KG Katsichti P Improvement of vascular endothelial function using the oral endothelin receptor antagonist bosentan in patients with systemic sclerosis. Arthritis Rheum 2007;56:1985–93 17530638 10.1002/art.22634

[22] Zouki C Baron C Fournier A Filep JG Endothelin-1 enhances neutrophil adhesion to human coronary artery endothelial cells: role of ET(A) receptors and platelet-activating factor. Br J Pharmacol 1999;127:969–79 10433505 10.1038/sj.bjp.0702593 PMC1566081

[23] Xu SW Denton CP Dashwood MR Abraham DJ Black CM Endothelin-1 regulation of intercellular adhesion molecule-1 expression in normal and sclerodermal fibroblasts. J Cardiovasc Pharmacol 1998;31 (Suppl 1):S545–7 9595538 10.1097/00005344-199800001-00157

[24] Waters CE Shi-Wen X Denton CP Abraham DJ Pearson JD Signaling pathways regulating intercellular adhesion molecule 1 expression by endothelin 1: comparison with interleukin-1β in normal and scleroderma dermal fibroblasts. Arthritis Rheum 2006;54:649–60 16447227 10.1002/art.21572

